# Haikun Shenxi Capsule Alleviates Renal Fibrosis in CKD Mice by Targeting the METTL3/IGF2BP3‐Mediated m6A Modification of NKD2

**DOI:** 10.1155/mi/4365859

**Published:** 2026-08-22

**Authors:** Mingjia Gu, Le Yang, Anjing Xu, Neng Bao, Ying Ni, Fang Cao, Xuejing Gu, Kejian Wen, Xiaolan Cheng

**Affiliations:** ^1^ Department of Nephrology, Changshu Hospital Affiliated to Nanjing University of Chinese Medicine, 6 Huanghe Road, Changshu 215500, Jiangsu, China; ^2^ Department of Basic Medicine, Wuxi School of Medicine, Jiangnan University, Wuxi 214122, Jiangsu, China, jiangnan.edu.cn; ^3^ Department of Nephrology, Affiliated Hospital of Jiangnan University, 1000 He Feng Road, Wuxi City 214000, Jiangsu, China, jiangnan.edu.cn; ^4^ School of Pharmacy, Ningxia Medical University, 1160 Shengli Street, Yinchuan 750004, China, nxmu.edu.cn; ^5^ School of Integrated Chinese and Western Medicine, Nanjing University of Chinese Medicine, Nanjing 210023, Jiangsu, China, njucm.edu.cn

**Keywords:** chronic kidney disease, Haikun Shenxi capsule, m6A, METTL3, NKD2

## Abstract

**Background:**

Renal fibrosis is a central pathological process in chronic kidney disease (CKD). Although Haikun Shenxi Capsule (HKSX) has shown clinical efficacy in CKD, its precise molecular targets and mechanisms remain unclear.

**Objective:**

This study aims to determine whether HKSX exerts antifibrotic effects in CKD by modulating NKD2 expression via METTL3‐mediated m6A pathway.

**Methods:**

A CKD mouse model was established by unilateral ureteral obstruction (UUO). Mice were divided into Sham, Model, low‐dose HKSX, high‐dose HKSX, and positive drug groups. Renal pathology and fibrosis were assessed by H&E, Masson’s trichrome, and Sirius red staining. Renal function was evaluated by measuring serum creatinine (Scr) and blood urea nitrogen (BUN) levels. Inflammatory cytokines, oxidative stress markers, apoptosis‐related proteins, fibrotic markers, NKD2, and METTL3 were detected by ELISA, qPCR, and Western blotting. Global m6A levels and NKD2 mRNA‐specific m6A enrichment were measured by colorimetric assay and MeRIP‐qPCR, respectively. Renal‐specific METTL3‐overexpressing mice were generated via lentivirus to validate target dependency. The role of the m6A reader IGF2BP3 was investigated using RIP‐qPCR.

**Results:**

HKSX significantly improved renal function and attenuated renal pathological injury, inflammation, oxidative stress, apoptosis, and fibrosis in UUO mice. Mechanistically, HKSX dose‐dependently downregulated METTL3 expression, reduced both global renal m6A levels and NKD2 mRNA‐specific m6A modification, and consequently inhibiting NKD2 overexpression. The protective effects of HKSX were largely abrogated in METTL3‐overexpressing CKD mice. Furthermore, HKSX specifically inhibited the expression of the m6A reader IGF2BP3 and its binding to NKD2 mRNA.

**Conclusion:**

This study demonstrates that HKSX alleviates renal fibrosis by inhibiting METTL3, which reduces m6A modification on NKD2 mRNA, and by downregulating the stability reader IGF2BP3. These effects collectively decrease NKD2 mRNA stability and protein expression. These findings reveal a novel epitranscriptomic mechanism of HKSX and identify the METTL3/IGF2BP3/NKD2 axis as a key therapeutic pathway in CKD.

## 1. Introduction

Chronic kidney disease (CKD) is a major public health concern characterized by structural abnormalities and progressive loss of renal function. It arises from various etiologies such as chronic glomerulonephritis and diabetic nephropathy. In recent years, the prevalence of CKD has continued to rise among the elderly population, while its onset is increasingly observed in younger individuals, imposing a substantial economic burden on society [1–4]. Renal fibrosis is the common pathological pathway in the progression of CKD to end‐stage renal disease, with its hallmark feature being the excessive deposition of extracellular matrix (ECM). ECM is primarily composed of collagen and fibronectin (FN), among other components [5]. Consequently, inhibiting aberrant ECM accumulation and retarding renal fibrosis are crucial therapeutic strategies for CKD.

The pathogenesis of renal fibrosis is intricately regulated by multiple signaling pathways. Among these, aberrant activation of the Wnt/β‐catenin signaling pathway plays a pivotal role. Upon kidney injury, this pathway is activated, leading to the nuclear translocation of β‐catenin and subsequent transcription of a series of target genes, including Wnt1. This process drives the transformation of renal interstitial cells into activated myofibroblasts, ultimately promoting massive ECM production [6]. NKD2, a key upstream regulator of this pathway, has recently been found to be highly expressed in various fibrotic diseases. Studies indicate that NKD2 serves as a specific marker for myofibroblasts [7] and is significantly upregulated in the kidneys of CKD model mice induced by unilateral ureteral obstruction (UUO) [8, 9]. These findings suggest that NKD2 may be a critical molecular link in the process of renal fibrosis.

However, the regulatory mechanisms governing NKD2 expression in CKD remain incompletely understood. Emerging evidence highlights the importance of epitranscriptomic modifications, particularly N6‐methyladenosine (m6A), in the progression of fibrotic diseases. m6A is the most prevalent internal modification in eukaryotic mRNA, and its dynamic equilibrium is maintained by methyltransferases (e.g., METTL3), demethylases, and reader proteins [10]. Dysregulated m6A modification has been shown to promote renal fibrosis by stabilizing fibrotic gene transcripts, enhancing the translation of profibrotic factors, or altering the expression of key signaling molecules. Emerging evidence indicates widespread dysregulation of m6A modification during CKD pathogenesis, wherein aberrant hypermethylation mediated by the methyltransferase METTL3 is a significant mechanism driving disease progression [11]. Given that NKD2 is a critical profibrotic molecule, we therefore hypothesize that NKD2 expression may be precisely regulated by METTL3‐mediated m6A modification, and this epigenetic mechanism could underlie its elevated expression in CKD and subsequent contribution to renal fibrosis.

Haikun Shenxi Capsule (HKSX) is a clinically approved Chinese patent medicine for CKD treatment, whose primary active ingredient is fucoidan. Extensive clinical practice has confirmed its efficacy in managing CKD [12], and preclinical studies have preliminarily revealed its potential for renal protection and antifibrotic effects [13]. Recent research further demonstrates that fucoidan effectively ameliorates renal fibrosis in CKD mice and suppresses TGF‐β‐induced fibrosis in renal tubular epithelial (HK‐2) cells [14, 15]. However, the specific molecular targets through which HKSX exerts its renoprotective effects, and whether it intervenes in renal fibrosis by regulating m6A modification, remain to be systematically and thoroughly investigated. In this study, utilizing a UUO mouse model, we aim to systematically elucidate the molecular mechanisms underlying the antirenal fibrotic effects of HKSX from the perspective of m6A modification, thereby providing a solid theoretical foundation for its clinical application.

## 2. Materials and Methods

### 2.1. Animals and Model Establishment

#### 2.1.1. Experimental Animals

Male C57BL/6J mice (6–8 weeks old) were supplied by GemPharmatech Co., Ltd. (Nanjing, China). All mice were housed under standard specific pathogen‐free (SPF) conditions with ad libitum access to food and water. The animal experimental protocol was approved by the Animal Care and Use Committee of Jiang Nan University (Approval No. JN. No20240515t0081130 [201]).

#### 2.1.2. CKD Model Establishment

The CKD model was established via UUO surgery. Briefly, mice were anesthetized by intraperitoneal injection of 50 mg/kg pentobarbital sodium, placed in a prone position, and a left flank incision was made. The left ureter was isolated and doubly ligated with silk sutures, then transected between the two ligation points. The incision was closed layer by layer. Mice in the Sham group underwent ureter isolation without ligation.

#### 2.1.3. Grouping and Drug Administration

Mice were randomly assigned to the following groups (*n* = 8 per group): Sham, Model, low‐dose HKSX group (100 mg/kg, HKSX‐L), high‐dose HKSX group (200 mg/kg, HKSX‐H) [16], and positive drug group (Losartan, 10 mg/kg/day) [17], Losartan (brand name: Cozaar, H20000371) was purchased from Hangzhou MSD Pharmaceutical Co., Ltd., with each tablet containing 50 mg. HKSX (manufactured by China Jilin Huinan Changlong Co., Ltd.) was dissolved in distilled water or sodium carboxymethyl cellulose (CMC‐Na) solution. Intragastric administration commenced on the second day postmodeling, once daily for 30 consecutive days. The Sham and Model groups received an equivalent volume of the vehicle.

### 2.2. Renal‐Specific METTL3 Overexpression Mouse Model

Renal‐specific METTL3 overexpression was achieved by tail vein injection of an adeno‐associated virus (AAV9) carrying METTL3 cDNA under the control of the kidney‐specific podocin promoter (Nphs1). The plasmid expressing METTL3 under the Nphs1 promoter was constructed and packaged into AAV9 (AAV9‐Nphs1‐METTL3). Mice were injected via the tail vein with a dose of 1 × 10^11^ vector genomes (vg) per mouse. Overexpression efficiency was assessed 3 weeks postinjection by Western blot and immunofluorescence (IF). Subsequently, both wild‐type (WT) and overexpression (OE METTL3) mice underwent UUO surgery and HKSX intervention. The mice were divided into the following groups: WT‐control, WT‐model, WT‐HKSX, OE METTL3‐control, OE METTL3‐model, and OE METTL3‐HKSX.

### 2.3. Sample Collection

After the final administration, blood was collected from mice, centrifuged to separate serum, and stored at −80°C for renal function assays. Mice were euthanized, and both kidneys were rapidly excised and weighed. Portions of renal tissue were fixed in 4% paraformaldehyde for histological examination, while other portions were immediately snap‐frozen in liquid nitrogen and subsequently transferred to −80°C for molecular biology analyses.

### 2.4. Histopathological Analysis

H&E, Masson’s trichrome, and Sirius red staining were performed according to standard operating procedures. Renal histomorphological changes and collagen fiber deposition were observed under a light microscope. The fibrosis area in Masson’s trichrome and Sirius red‐stained sections was quantified using Image‐Pro Plus or ImageJ software. For immunohistochemistry (IHC), paraffin‐embedded sections were deparaffinized, rehydrated, and subjected to antigen retrieval. Sections were incubated overnight at 4°C with primary antibodies against CD68, Collagen I, NKD2, etc. This was followed by incubation with corresponding HRP‐conjugated secondary antibodies and development using a DAB chromogen kit, with hematoxylin counterstaining for nuclei. Staining was observed and imaged under a microscope, and semiquantitative analysis of positive expression areas was performed.

### 2.5. Renal Function Measurement

Commercial kits were used according to the manufacturer’s instructions to measure serum urea nitrogen (BUN) and serum creatinine (Scr) levels using a fully automated biochemical analyzer.

### 2.6. Enzyme‐Linked Immunosorbent Assay (ELISA)

Portions of renal tissue were homogenized, centrifuged, and the supernatant was collected. Specific ELISA kits for mouse IL‐1β, IL‐6, TNF‐α, and IL‐10 were used to detect the protein concentrations of these inflammatory cytokines strictly following the kit protocols.

### 2.7. RNA Extraction and Real‐Time Quantitative PCR (RT‐qPCR)

Total RNA was extracted from kidney tissues using the TRIzol method, and its concentration and purity were determined. One microgram of total RNA was reverse‐transcribed into first‐strand cDNA using a reverse transcription kit. Using cDNA as a template, amplification was performed on a real‐time PCR system with SYBR Green Premix. The relative mRNA expression levels of target genes (IL‐1β, IL‐6, TNF‐α, IL‐10, Collagen I, Collagen III, FN, E‐cadherin, NKD2, METTL3, YTHDF1/2/3, and IGF2BP1/2/3) were calculated using the 2^−ΔΔ*Ct*^ method, with GAPDH as the internal reference.

### 2.8. Protein Extraction and Western Blot Analysis

Total protein was extracted from kidney tissues using RIPA lysis buffer containing protease and phosphatase inhibitors. Protein concentration was determined by the BCA method. Equal amounts of protein were separated by SDS‐PAGE and transferred to PVDF membranes. After blocking with 5% skim milk, membranes were incubated overnight at 4°C with specific primary antibodies (e.g., against IL‐1β, 12‐LOX, Bax, Bcl‐2, Cleaved‐caspase3, Caspase3, NKD2, METTL3, p‐Smad3, Smad3, COL1A1, α‐SMA, E‐cadherin, β‐actin). After washing, membranes were incubated with corresponding HRP‐conjugated secondary antibodies for 1 h at room temperature. Signals were visualized using an ECL chemiluminescence kit and captured using a chemiluminescence imaging system. Band intensity was quantified using ImageJ software, and relative expression was represented as the ratio of target protein to internal reference (GAPDH).

### 2.9. Detection of m6A Modification

For global m6A level measurement, an m6A RNA methylation quantification kit (colorimetric method) was used strictly according to the manufacturer’s instructions. Absorbance was measured using a microplate reader to calculate the relative m6A content in total RNA from kidney tissue.

An m6A methylated RNA immunoprecipitation (MeRIP) kit was used to detect the NKD2 mRNA‐specific m6A level. Briefly, total RNA was fragmented. A portion was saved as Input control, while another portion was incubated overnight at 4°C with an anti‐m6A antibody or isotype IgG antibody. Antibody–RNA complexes were captured using protein A/G magnetic beads, followed by elution and purification of the co‐precipitated RNA. Finally, NKD2 mRNA levels in both Input and IP samples were detected by RT‐qPCR. The relative m6A enrichment of NKD2 mRNA was expressed as the IP/Input ratio.

### 2.10. RNA Immunoprecipitation (RIP)‐qPCR

Kidney tissue homogenates were lysed, and a portion of the lysate was saved as Input. The remaining lysate was incubated overnight at 4°C with anti‐IGF2BP3 antibody or normal IgG antibody coupled with protein A/G magnetic beads. After serial washes, the RNA bound to the beads was eluted and purified. NKD2 mRNA levels in Input and IP samples were detected by RT‐qPCR. The binding level of IGF2BP3 to NKD2 mRNA was expressed as the IP/Input ratio.

### 2.11. IF and Double IF Labeling

Frozen or paraffin sections were fixed, permeabilized, and blocked, then incubated overnight at 4°C with primary antibodies (e.g., against NKD2, METTL3, m6A, COL1A1, p‐Smad3, α‐SMA). After washing, sections were incubated with corresponding fluorophore‐conjugated (e.g., FITC, Cy3) secondary antibodies for 1 h at room temperature in the dark. Nuclei were counterstained with DAPI. Images were observed and captured using a fluorescence microscope or confocal microscope. For double labeling, two primary antibodies from different hosts and two corresponding secondary antibodies conjugated with different fluorophores were used.

### 2.12. Statistical Analysis

All data are presented as mean ± standard deviation. Statistical analysis was performed using GraphPad Prism software. Comparisons among multiple groups were analyzed by one‐way analysis of variance (ANOVA) followed by Tukey’s post hoc test. Before performing one‐way ANOVA, the normality of distribution was assessed using the Shapiro–Wilk test, and homogeneity of variances was evaluated using Levene’s test. A *P* value < 0.05 was considered statistically significant.

## 3. Results

### 3.1. Effects of HKSX on Renal Gross Morphology, Histopathology, and Fibrosis in CKD Mice

**Figure 1:**
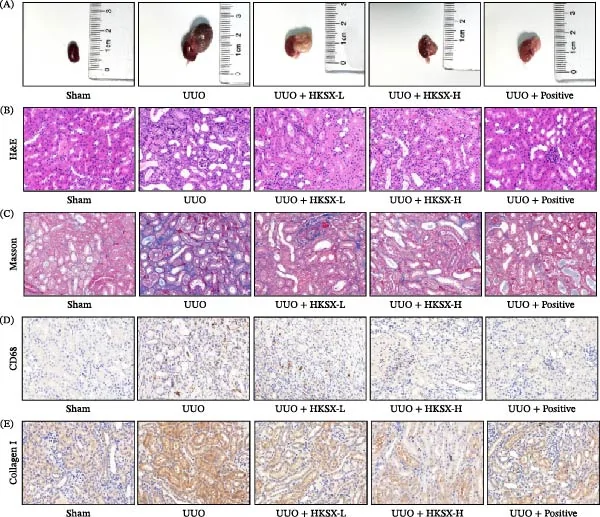
HKSX ameliorates renal histopathological injury and fibrosis in UUO‐induced CKD mice. (A) Representative gross morphology of kidneys from each group. (B) Representative images of H&E staining showing renal histopathology. (C) Representative images of Masson’s trichrome staining showing collagen deposition (blue). (D,E) Representative immunohistochemical staining images and quantitative analysis for (D) CD68 (macrophage marker), and (E) Collagen I.

To evaluate the therapeutic effects of HKSX on CKD, we first examined renal gross morphology and histopathological changes. As shown in Figure 1A, kidneys from the Sham group appeared dark red with smooth surfaces. In contrast, the Model group exhibited significantly enlarged kidneys with rough surfaces, indicating severe inflammatory edema and injury. Treatment with HKSX at both low and high doses (HKSX‐L and HKSX‐H) ameliorated renal enlargement, with the HKSX‐H and positive control groups showing the most pronounced improvement.

H&E staining (Figure 1B) revealed normal glomerular and tubular structures in the Sham group. The Model group displayed typical CKD pathologies, including glomerular atrophy, tubular dilation, vacuolar degeneration, necrosis of tubular epithelial cells, and extensive inflammatory cell infiltration. These pathological changes were alleviated to varying degrees by positive control and HKSX treatments. Masson’s trichrome staining (Figure 1C) showed minimal collagen fibers in the Sham group, whereas the Model group exhibited extensive blue collagen deposition in the mesangium, peritubular areas, and interstitium. Both the positive control and HKSX treatments effectively reduced collagen deposition, with HKSX‐H showing efficacy comparable to that of the positive control.

IHC for CD68 (a macrophage marker) and Collagen I was performed to quantify inflammation and fibrosis. CD68 staining (Figure 1D) showed a significant increase in positive cells in the Model group, which was dose‐dependently reduced by HKSX. Collagen I staining (Figure 1E) confirmed excessive deposition in the Model group, which was effectively suppressed by both positive control and HKSX treatments.

### 3.2. Effects of HKSX on Renal Function and Fibrosis‐Related Molecules in CKD Mice

**Figure 2:**
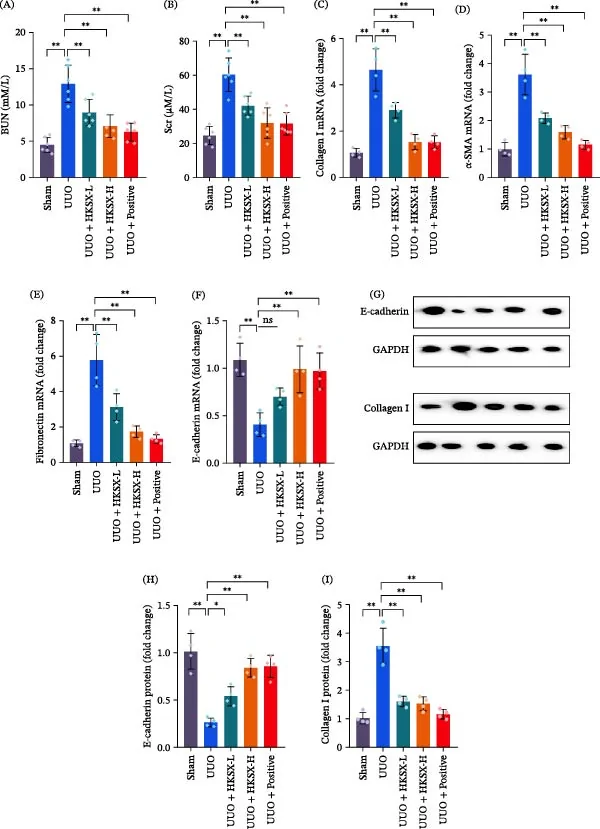
HKSX improves renal function and suppresses the expression of fibrosis‐related molecules in CKD mice. (A,B) Serum levels of (A) blood urea nitrogen (BUN) and (B) creatinine (Scr). (C–F) mRNA expression levels of (C) Collagen I, (D) α‐SMA, (E) Fibronectin, and (F) E‐cadherin in renal tissues as determined by RT‐qPCR. (G) Representative Western blot bands for Collagen I and E‐cadherin. (H,I) Quantitative analysis of (H) E‐cadherin and (I) Collagen I protein levels normalized to GAPDH.

Renal function parameters and the expression of key fibrotic genes and proteins were assessed. Serum BUN and creatinine (Scr) levels were significantly elevated in the Model group, indicating severe renal impairment (Figure 2A, B). HKSX treatment, particularly HKSX‐H, effectively reduced BUN and Scr levels, showing efficacy similar to that of the positive control.

RT‐qPCR analysis (Figure 2C–F) showed that, compared with the Sham group, the Model group had significantly upregulated mRNA levels of pro‐fibrotic genes (Collagen I, α‐SMA, FN) and downregulated E‐cadherin, indicating epithelial‐mesenchymal transition (EMT) activation. HKSX treatment dose‐dependently reversed these changes. Western blot analysis (Figure 2G–I) confirmed these findings at the protein level. HKSX significantly inhibited Collagen I protein expression and promoted E‐cadherin protein expression, demonstrating its antifibrotic effect via suppression of ECM deposition and EMT.

### 3.3. Effects of HKSX on Renal Inflammation, Oxidative Stress, and Apoptosis in CKD Mice

To investigate the mechanisms underlying HKSX’s renal protective effects in CKD mice, we detected expression levels of inflammatory factors, oxidative stress, and apoptosis‐related indicators in renal tissue using ELISA, qPCR, and Western blotting. Consistent results from ELISA and qPCR demonstrated significantly elevated protein and mRNA levels of IL‐1β, IL‐6, and TNF‐α, along with a marked reduction in IL‐10 in Model group compared to the Sham group (Figure 3A–H). Compared with the Model group, the HKSX‐L and HKSX‐H groups dose‐dependently reduced IL‐1β, IL‐6, and TNF‐α levels while significantly enhancing IL‐10 expression.

Figure 3HKSX alleviates renal inflammation, oxidative stress, and apoptosis in CKD mice. (A–D) Protein levels of inflammatory cytokines (A) IL‐1β, (B) IL‐6, (C) TNF‐α, and (D) IL‐10 in renal tissues measured by ELISA. (E–H) mRNA expression levels of (E) IL‐1β, (F) IL‐6, (G) TNF‐α, and (H) IL‐10 determined by RT‐qPCR. (I–M) Representative Western blot bands and quantitative analysis for IL‐1β (I), 12‐LOX (J), Bax (K), Bcl‐2 (L), and Cleaved‐caspase 3 (M). Data are presented as mean ± SD. Normality and homogeneity of variances were verified using the Shapiro‐Wilk test and Levene’s test, respectively; all datasets satisfied the assumptions for ANOVA (*p* > 0.05). Multiple comparisons were adjusted using Tukey’s post hoc test following one‐way ANOVA.  ^∗^ *p* < 0.05,  ^∗∗^ *p* < 0.01.

**Figure:**
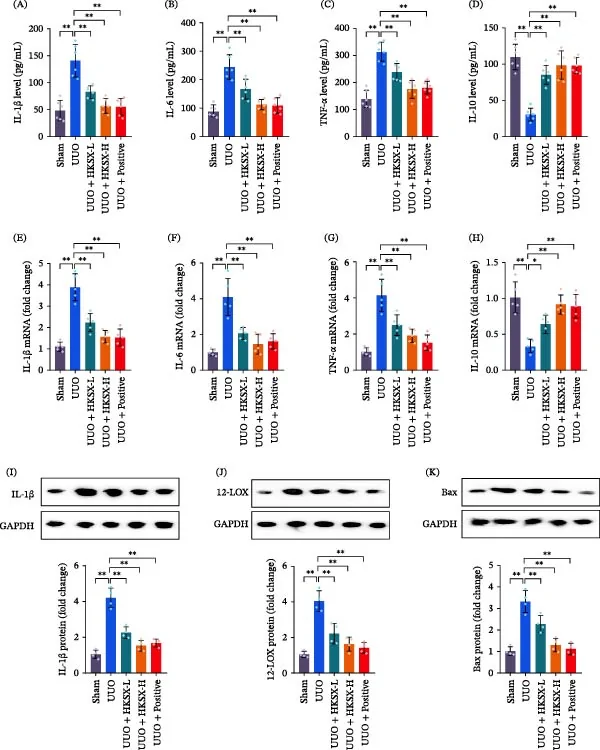

**Figure:**
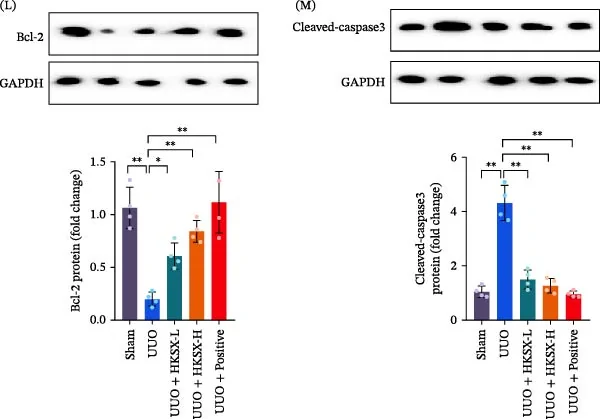

Western blot results further verified HKSX’s anti‐inflammatory effects. As shown in Figure 3I, J, IL‐1β and 12‐LOX were significantly upregulated in the Model group compared with the Sham group. HKSX treatment induced a dose‐dependent reduction in IL‐1β and 12‐LOX. Furthermore, the Model group exhibited significantly increased expression of Bax and cleaved caspase‐3 (activated form of caspase‐3), along with markedly decreased expression of the antiapoptotic protein Bcl‐2 (Figure 3K–M). HKSX treatment effectively inhibited the expression of these apoptosis‐related proteins.

### 3.4. HKSX Inhibited NKD2 Expression via METTL3‐Mediated m6A Modification

**Figure 4:**
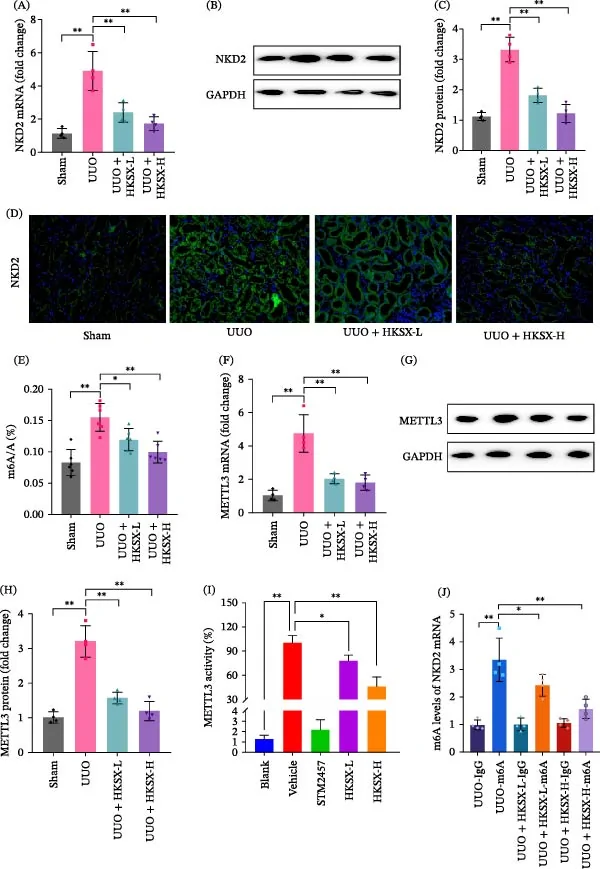
HKSX inhibits NKD2 expression by modulating METTL3‐mediated m6A modification. (A) mRNA expression level of NKD2 in kidney. (B) Representative Western blot bands for NKD2. (C) Quantitative analysis of NKD2 protein level. (D) Representative immunofluorescence staining images for NKD2 (green). Nuclei are stained with DAPI (blue). (E) Global m6A levels in total renal RNA. (F) mRNA expression level of METTL3. (G) Representative Western blot bands for METTL3. (H) Quantitative analysis of METTL3 protein level. (I) Analysis of METTL3 enzymatic activity. (J) m6A enrichment on NKD2 mRNA measured by MeRIP‐qPCR. Data are presented as mean ± SD. Normality and homogeneity of variances were verified using the Shapiro–Wilk test and Levene’s test, respectively; all datasets satisfied the assumptions for ANOVA (*p* > 0.05). Multiple comparisons were adjusted using Tukey’s post hoc test following one‐way ANOVA.  ^∗^ *p* < 0.05,  ^∗∗^ *p* < 0.01.

Aberrant NKD2 activation is closely associated with CKD progression, particularly renal tubulointerstitial fibrosis. To investigate the molecular mechanisms of HKSX’s renal protection, we detected NKD2 expression using qPCR, Western blotting, and IF. Consistent qPCR and Western blot results (Figure 4A–C) demonstrated significantly upregulated NKD2 mRNA and protein levels in the Model group compared with the Sham group, confirming excessive NKD2 activation in CKD model mice. Compared with the Model group, HKSX‐L and HKSX‐H dose‐dependently inhibited NKD2 overexpression. IF staining further validated these results (Figure 4D). The Model group exhibited strong NKD2 expression in renal cells. HKSX administration markedly reduced NKD2 fluorescence intensity, indicating effective suppression of NKD2 activation by HKSX.

m6A represents the most prevalent epigenetic modification in eukaryotic mRNA, with its writer protein METTL3 being aberrantly expressed in various renal diseases. To investigate whether HKSX regulates NKD2 expression at the epigenetic level, we detected global m6A levels, METTL3 expression, and NKD2 mRNA‐specific m6A modification levels. Colorimetric assay results (Figure 4E) showed significantly elevated total renal m6A levels in the Model group compared to the Sham group, indicating extensive m6A methylation abnormalities in the CKD model. Subsequent qPCR and Western blot detection of core m6A methyltransferase METTL3 expression yielded results consistent with global m6A level changes (Figure 4F–H), showing significant upregulation of METTL3 mRNA and protein in the Model group. HKSX treatment effectively reversed this trend, dose‐dependently reducing total m6A levels and downregulating METTL3 expression.

To further determine whether HKSX directly inhibits METTL3 enzymatic activity or merely downregulates its expression, we performed an in vitro methyltransferase activity assay using recombinant METTL3/METTL14 complex treated with HKSX‐medicated serum (Supporting Information). As shown in Figure 4I, HKSX‐medicated serum significantly reduced METTL3 methyltransferase activity in a dose‐dependent manner, suggesting that HKSX directly suppresses METTL3 catalytic function. To directly verify whether NKD2 is regulated by m6A modification, MeRIP‐qPCR was employed to specifically detect m6A enrichment on NKD2 mRNA. As shown in Figure 4J, NKD2 mRNA m6A modification levels were significantly increased in the Model group compared to Sham. HKSX treatment significantly reduced NKD2 mRNA m6A levels. These findings suggest that HKSX may suppress NKD2 expression by inhibiting METTL3 expression and consequently reducing m6A modification on NKD2 mRNA.

### 3.5. METTL3 Overexpression Abolishes HKSX‐Mediated Renoprotection in CKD Mice

To verify whether HKSX exerts its therapeutic effects through METTL3 inhibition, we generated renal‐specific METTL3‐overexpressing transgenic mice and established renal injury models via UUO surgery. Western blotting and IF confirmed significantly higher METTL3 protein levels in the kidneys of overexpression (OE METTL3) mice compared with WT mice (Figure S1), validating successful model establishment.

As shown in Figure 5A, results from H&E, Masson, and Sirius red staining revealed that, compared with the WT‐control group, the WT‐model group exhibited severe tubular dilation, inflammatory cell infiltration, and significant collagen fiber deposition (blue/red). WT‐HKSX treatment markedly ameliorated these pathological changes, significantly reducing tubular structural damage and interstitial fibrosis area. In METTL3‐overexpressing mice, the OE METTL3‐control group showed no significant pathological alterations. However, the OE METTL3‐model group demonstrated more severe renal injury and fibrosis than the WT‐model group. Crucially, under METTL3 overexpression condition, although the OE METTL3‐HKSX group showed some reduction in fibrosis area compared with the OE METTL3‐model group, it failed to recover to the level of improvement seen in the WT‐HKSX group (Figure 5B, C). This indicates that METTL3 overexpression significantly attenuates HKSX’s antifibrotic efficacy.

Figure 5METTL3 overexpression abolishes the renoprotective and antifibrotic effects of HKSX. (A) Representative images of H&E, Masson’s trichrome, and Sirius red staining of kidney sections from the indicated groups. (B,C) Quantitative analysis of fibrosis area from (B) Masson’s trichrome and (C) Sirius red staining. (D–G) Representative immunofluorescence staining images and quantitative analysis for (D,E) α‐SMA and (F,G) COL1A1. (H) Representative Western blot bands for α‐SMA and COL1A1. (I,J) Quantitative analysis of (I) COL1A1, and (J) α‐SMA protein levels. Data are presented as mean ± SD, *n* = 3.

**Figure:**
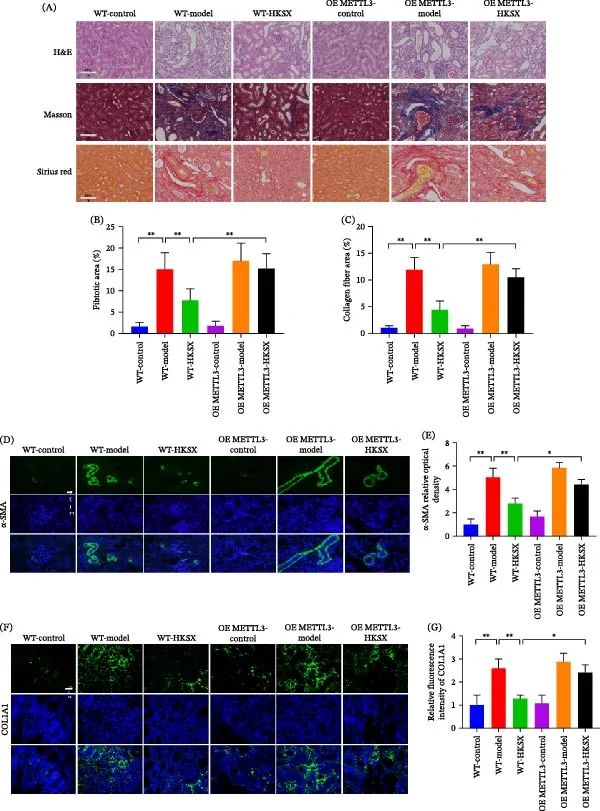

**Figure:**
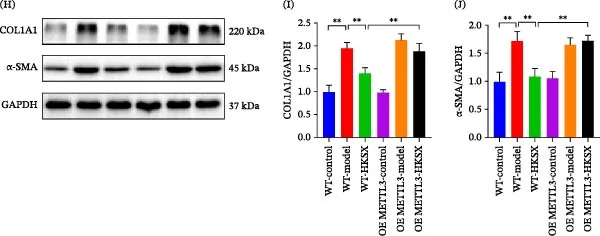

Further IF and Western blot detection of key fibrotic markers (Figure 5D–J) showed that UUO significantly upregulated myofibroblast marker α‐SMA and collagen protein COL1A1. HKSX effectively suppressed α‐SMA and COL1A1 upregulation in the WT‐model group. However, in METTL3‐overexpressing mice, the inhibitory effects of HKSX on α‐SMA and COL1A1 were significantly weakened. These results confirm that METTL3 overexpression not only exacerbates UUO‐induced renal fibrosis but also the renoprotective and antifibrotic effects of HKSX.

### 3.6. METTL3 Overexpression Antagonizes HKSX Effects by Enhancing NKD2 and Activating the TGF‐β/Smad Pathway

**Figure 6:**
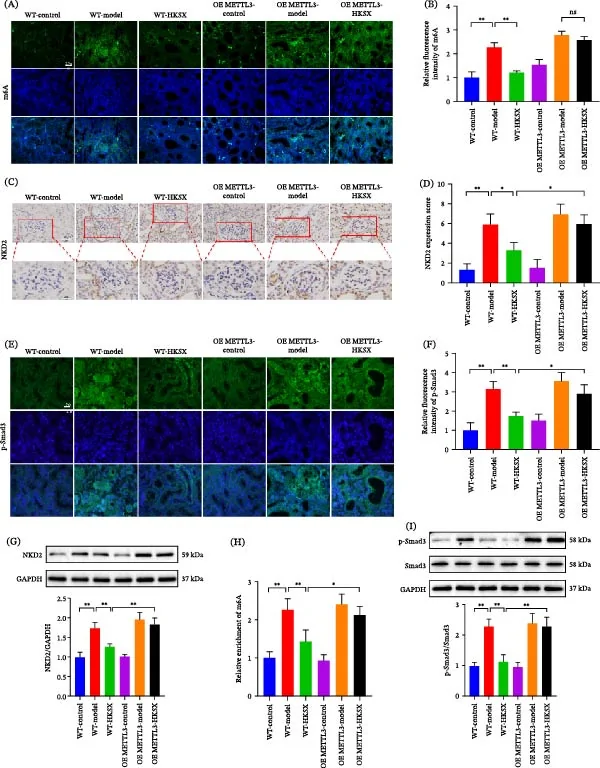
METTL3 overexpression antagonizes HKSX effects by sustaining NKD2 m6A modification and activating profibrotic signaling. (A,B) Representative immunofluorescence staining images (A) and quantitative analysis (B) for global m6A levels. (C,D) Representative immunohistochemical staining images and quantitative analysis for NKD2. (E,F) Representative immunofluorescence staining images (E) and quantitative analysis (F) for p‐Smad3. (G) Representative Western blot bands and quantitative analysis for NKD2. (H) NKD2 mRNA m6A enrichment, (I) Representative Western blot bands and quantitative analysis for p‐Smad3/Smad3. Data are presented as mean ± SD, *n* = 3.

To elucidate the molecular mechanism by which METTL3 overexpression impairs HKSX efficacy, we focused on the m6A‐NKD2 axis and its downstream signaling pathways. IF detection (Figure 6A, B) showed significantly enhanced global renal m6A fluorescence intensity in the WT‐model group, which was reduced by WT‐HKSX treatment. In the OE METTL3‐model group, the m6A signal was strongest, and the OE METTL3‐HKSX group maintained high levels, indicating that METTL3 overexpression sustains a high m6A state in the kidneys.

IHC and Western blot results (Figure 6C, D,G) revealed that in WT mice, HKSX significantly downregulated NKD2, whereas in OE METTL3 mice, both basal and HKSX‐induced NKD2 expression were strongly impaired. NKD2 protein levels in the OE METTL3‐HKSX group were significantly higher than those in the WT‐HKSX group. MeRIP‐qPCR‐specific detection of NKD2 mRNA m6A enrichment demonstrated that, in WT CKD mice, HKSX intervention significantly reversed NKD2 mRNA hypermodification. NKD2 m6A levels in the OE METTL3‐HKSX group were significantly higher than those in the WT‐HKSX group and showed no significant difference from those in the OE METTL3‐model group (Figure 6H).

The TGF‐β/Smad pathway is a core driver of renal fibrosis. Detection of phosphorylated Smad3 (p‐Smad3, the activated form of this pathway) via IF and Western blot (Figure 6E, F,I) showed HKSX inhibited p‐Smad3 elevation in the WT‐model group. However, in the OE METTL3‐model group, p‐Smad3 levels were extremely high, and in the OE METTL3‐HKSX group, p‐Smad3 levels were not effectively reduced by HKSX, remaining significantly higher than those in the WT‐HKSX group. This trend paralleled the changes in COL1A1 expression.

### 3.7. HKSX Reduces NKD2 mRNA Stability via the m6A Reader IGF2BP3

To elucidate how HKSX downregulates NKD2 protein expression following the reduction of its m6A modification, we investigated the role of m6A reader proteins in this process. The functional outcome of m6A modification depends on recognition by specific readers: YTHDF family proteins typically mediate mRNA degradation, whereas IGF2BP family proteins enhance mRNA stability and translation.

**Figure 7:**
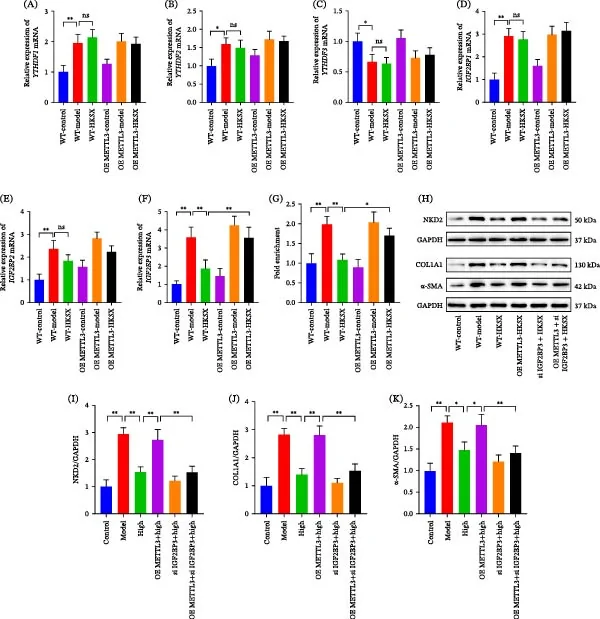
HKSX reduces NKD2 mRNA stability via an m6A reader IGF2BP3‐dependent mechanism. (A–F) mRNA expression levels of m6A reader proteins: (A) YTHDF1, (B) YTHDF2, (C) YTHDF3, (D) IGF2BP1, (E) IGF2BP2, and (F) IGF2BP3. (G) Binding of IGF2BP3 to NKD2 mRNA assessed by RIP‐qPCR. (H) Representative Western blot bands for NKD2, COL1A1 and α‐SMA. (I–K) Quantitative analysis of (I) NKD2, (J) COL1A1 and (K) α‐SMA protein levels. Data are presented as mean ± SD, *n* = 3.

Initial qPCR detection of major m6A reader mRNA levels (Figure 7A–F) showed that, compared with the Sham group, the Model group exhibited significant upregulation of YTHDF1, YTHDF2, IGF2BP1, IGF2BP2, and IGF2BP3. HKSX intervention demonstrated selective regulatory effects. Compared with the Model group, HKSX‐H significantly suppressed IGF2BP3 mRNA expression but showed no significant effects on YTHDF2, IGF2BP1, or IGF2BP2 expression. This suggests HKSX may determine the fate of m6A‐modified NKD2 mRNA by specifically inhibiting IGF2BP3 expression.

To directly verify IGF2BP3 binding to NKD2 mRNA, RNA immunoprecipitation (RIP)‐qPCR was performed. Results (Figure 7G) showed significantly enhanced IGF2BP3 protein binding to NKD2 mRNA in Model group compared to Sham. HKSX‐H treatment significantly reduced IGF2BP3 binding to NKD2 mRNA. Thus, HKSX decreases NKD2 by both reducing global m6A levels and specifically inhibiting IGF2BP3 binding to NKD2 mRNA.

To further dissect the hierarchical relationship between METTL3 and IGF2BP3 in mediating the antifibrotic effect of HKSX, we performed IGF2BP3 knockdown experiments in BUMPT cells treated with HKSX‐medicated serum, with or without METTL3 overexpression (Supporting Information). As shown in Figure 7H–K, IGF2BP3 knockdown alone significantly reduced the protein levels of fibrotic markers COL1A1 and α‐SMA, as well as decreasing NKD2 expression, phenocopying the effects of HKSX treatment. METTL3 overexpression markedly attenuated the inhibitory effects of HKSX on these fibrotic markers. However, when IGF2BP3 knockdown was combined with METTL3 overexpression, the inhibitory effect of HKSX was restored, as evidenced by reduced COL1A1, α‐SMA, and NKD2 protein levels compared to METTL3 overexpression alone (Figure 7H–K). These results demonstrate that IGF2BP3 acts downstream of METTL3, and that HKSX exerts its antifibrotic effect by inhibiting the METTL3/IGF2BP3 axis.

## 4. Discussion

This study is the first to elucidate the molecular mechanism by which HKSX exerts its antifibrotic effects in CKD via METTL3‐mediated m6A modification and subsequent NKD2 suppression. Our core finding is that HKSX inhibited both the expression and enzymatic activity of METTL3, thereby reducing global and NKD2 mRNA‐specific m6A modification, ultimately blocking the pro‐fibrotic factor NKD2. Functional rescue experiments confirmed that, in renal‐specific METTL3‐overexpressing mice, the renal protective effects of HKSX and its regulation on NKD2 were significantly attenuated, establishing METTL3 as a central target of HKSX efficacy. Mechanistically, downstream of METTL3, HKSX specifically suppressed the m6A reader protein IGF2BP3 and its binding to NKD2 mRNA, disrupting NKD2 mRNA stability and reducing its translational efficiency. Notably, using genetic interaction experiments (IGF2BP3 knockdown with or without METTL3 overexpression), we demonstrated that IGF2BP3 acts downstream of METTL3, forming a linear METTL3/IGF2BP3/NKD2 axis—the core pathway through which HKSX exerts its antifibrotic effects.

Since its introduction into clinical use in 2003, the therapeutic efficacy of HKSX for CKD has been widely recognized. However, research into the underlying molecular mechanisms of HKSX remains insufficient. An early study by Zhao et al. [13] found that HKSX improved renal function and inhibited PDGF‐BB expression in adriamycin‐induced nephropathy rats but did not further explore its specific targets. Subsequent research has focused more on its active ingredient, fucoidan, demonstrating its ability to delay renal fibrosis progression by regulating the inflammasome [18], autophagy [19], and cytokine‐related pathways [20]. Consistent with the antifibrotic phenotype, we confirmed that HKSX significantly inhibited excessive ECM deposition (Collagen I, FN) and reversed E‐cadherin downregulation, indicating effective antagonism of EMT. Additionally, HKSX reduced 12‐LOX (oxidative stress), pro‐inflammatory cytokines (IL‐1β, IL‐6, and TNF‐α), and modulated the Bcl‐2/Bax ratio to inhibit apoptosis. These multifaceted protective effects of HKSX collectively improve renal function.

NKD2 is a myofibroblast marker that correlates with CKD severity [7]. Our research confirmed significantly elevated NKD2 levels in the kidneys of UUO‐induced CKD mice [21]. However, the post‐transcriptional regulation of NKD2 remained unclear. Here, we reveal that the pathological overexpression of NKD2 is profoundly influenced by m6A modification. Our data showed increased levels of both METTL3 and global m6A in the CKD model, consistent with reported references [22, 23]. More importantly, using MeRIP‐qPCR, we directly confirmed a specific increase in m6A modification on NKD2 mRNA in the Model group, which was reversed by HKSX treatment. Critically, in METTL3‐overexpressing CKD mice, HKSX failed to effectively reduce NKD2 m6A modification and protein expression and could not exert its antifibrotic effects, providing direct genetic evidence for the hypothesis that METTL3 is a core target of HKSX. Furthermore, our in vitro methyltransferase activity assay demonstrated that HKSX‐medicated serum directly suppressed the enzymatic activity of the recombinant METTL3/METTL14 complex in a dose‐dependent manner. This finding extends the mechanistic understanding of HKSX beyond transcriptional downregulation of METTL3, revealing that HKSX directly inhibits METTL3 catalytic function.

To further investigate how HKSX influences the fate downstream of m6A modification, we focused on m6A reader proteins. Among the various readers, HKSX specifically downregulated IGF2BP3, with little effect on the YTHDF family or other IGF2BP members. IGF2BP3 is known for enhancing target mRNA stability and translation efficiency by recognizing m6A modifications [24–26]. RIP‐qPCR experiments confirmed that enhanced binding between IGF2BP3 and NKD2 mRNA in the CKD model was significantly disrupted by HKSX. We thus propose that under pathological conditions, METTL3‐mediated hyper‐m6A modification of NKD2 mRNA provides ample binding sites for IGF2BP3, stabilizing NKD2 mRNA and promoting its translation, leading to elevated NKD2 protein levels and driving the fibrotic process. HKSX, by inhibiting METTL3, reduces the number of m6A “tags” while simultaneously downregulating IGF2BP3 expression. This dual action jointly weakens the m6A‐IGF2BP3 axis‐mediated stabilization of NKD2 mRNA. To dissect the hierarchy between METTL3 and IGF2BP3, we performed IGF2BP3 knockdown with or without METTL3 overexpression. IGF2BP3 knockdown alone phenocopied the antifibrotic effects of HKSX, whereas METTL3 overexpression attenuated HKSX efficacy. Notably, combined IGF2BP3 knockdown restored HKSX efficacy, indicating that IGF2BP3 acts downstream of METTL3.

Although this study systematically reveals a novel mechanism by which HKSX attenuates renal fibrosis via the METTL3/IGF2BP3/NKD2 axis, several limitations should be acknowledged. First, HKSX is a multi‐component traditional Chinese medicine formula. While this study identified the downstream targets of the overall action of HKSX, determining which specific active ingredients are primarily responsible for inhibiting the METTL3/IGF2BP3 axis requires further compound screening and validation. Second, all conclusions drawn in this study are based on preclinical animal models and molecular biology experiments. The clinical translational value of these findings needs verification in future clinical trials. Future studies will focus on isolating and identifying specific components within HKSX that target the METTL3/IGF2BP3 axis to develop more targeted antifibrotic therapies. In summary, this study not only provides a pharmacological mechanism for HKSX in treating CKD but also lays a scientific foundation for developing new strategies targeting m6A modification in kidney diseases.

## Author Contributions

Xiaolan Cheng and Mingjia Gu conceived and designed research. Le Yang, Anjing Xu, Fang Cao, Ying Ni, and Neng Bao conducted experiments. Xuejing Gu and Kejian Wen analyzed data. Mingjia Gu and Xiaolan Cheng wrote the manuscript.

## Funding

A joint support was given to this study by the China Association of Chinese Medicine Joint Research Project (Grant 2023DYPLHGG‐10), the National Natural Science Foundation of China (Grant 82474471), the Jiangsu Province Traditional Chinese Medicine Technology Development Plan Project (Grant MS2022085), the Suzhou Gusu Health Talent Program Research Project (Grant GSWS2022099), the Suzhou Science and Technology Development Project (Grant SYW2024010), and the Changshu Science and Technology Development Plan Project (Grant CS202458).

## Disclosure

All authors read and approved the manuscript. All data were generated in‐house, and no paper mill was used. All authors agree to be accountable for all aspects of work, ensuring integrity and accuracy.

## Conflicts of Interest

The authors declare no conflicts of interest.

## Supporting Information

Additional supporting information can be found online in the Supporting Information section.

## Supporting information


**Supporting Information** Figure S1: Overexpression efficiency of renal METTL3 overexpression was assessed by Western blot and IF. (A, B) Representative IF staining images (A) and quantitative analysis (B) for METTL3 overexpression. (C) Representative Western blot bands and quantitative analysis (D) for METTL3.

## Data Availability

The datasets used and analyzed during the current study are available from the corresponding author upon reasonable request.
