# Supplementary material for: Haikun Shenxi Capsule Alleviates Renal Fibrosis in CKD Mice by Targeting the METTL3/IGF2BP3‐Mediated m6A Modification of NKD2
Source: Mediators Inflamm. 2026 Aug 22;2026:4365859. doi: 10.1155/mi/4365859 (PMC13498966; doi:10.1155/mi/4365859)
Supplement: Supplementary file 1 [file MI-2026-4365859-s001.docx]

**METTL3/METTL14 methyltransferase activity assay**

The direct inhibitory effect of HKSX‑medicated serum on METTL3 catalytic activity was assessed using the METTL3/METTL14 Complex Chemiluminescent Assay Kit (BPS Biosciences, #79614), which detects N⁶‑methyladenosine generated on a pre‑coated RNA substrate via specific antibody‑based chemiluminescence. Serum was collected from rats administered low‑ (100 mg/kg) or high‑dose (100 mg/kg) HKSX, or saline (vehicle), heat‑inactivated, filtered, and stored at −80 °C. In vitro reactions contained recombinant METTL3/METTL14 complex (200 ng/well), S‑adenosylmethionine (SAM), and the RNA substrate, with the following groups: Blank (no enzyme, no serum), Vehicle (5 µL blank serum), STM2457 (5 µL blank serum containing 10 µM STM2457 as positive control), HKSX‑L (5 µL low‑dose medicated serum), and HKSX‑H (5 µL high‑dose medicated serum). All serum‑containing groups received equal total serum volume (5 µL) to maintain consistent matrix effects. After overnight incubation at room temperature, m⁶A levels were detected by sequential incubation with primary anti‑m⁶A antibody and HRP‑conjugated secondary antibody, followed by chemiluminescence measurement (RLU). Net RLU (after subtracting Blank) from the Vehicle group was set as 100% activity; relative activities and inhibition rates were calculated accordingly.

**Supplementary Materials and Methods**

**1. Cell Culture**

Mouse proximal tubular epithelial BUMPT cells were cultured in high-glucose Dulbecco’s Modified Eagle Medium (DMEM) supplemented with 10% fetal bovine serum (FBS) and 1% penicillin–streptomycin mixture (final concentrations: 100 U/mL penicillin and 100 μg/mL streptomycin). Cells were cultured at 37℃ in a humidified atmosphere containing 5% CO_2_.

**2. Preparation of HKSX-Medicated Rat Serum**

Eight-week-old male Sprague-Dawley (SD) rats weighing approximately 200 g were housed under specific pathogen-free (SPF) conditions with controlled ambient temperature (25±2°C) and a 12 h light/dark cycle. Standard diet and water were provided ad libitum. The daily intragastric administration dosage of HKSX was calculated according to the conversion formula for equivalent doses between humans and experimental animals. Intragastric gavage was performed twice daily at 9:00 a.m. and 4:00 p.m. for 7 consecutive days. Two hours after the final gavage, rats were anesthetized, and blood samples were collected via abdominal aortic puncture. Whole blood was centrifuged at 3000 r/min for 10 min to isolate the serum. The collected serum was heat-inactivated at 56°C for 30 min, sterile-filtered through a 0.22 μm microporous membrane, and preserved at −80 °C for subsequent cell treatments.

**3. Lentiviral-Mediated Establishment of METTL3-Overexpressing BUMPT Cells**

Recombinant lentiviral vectors overexpressing METTL3 were constructed, and lentiviral particles were generated using the third-generation lentiviral packaging system. Briefly, the lentiviral overexpression plasmid (lenti-OE-METTL3) and two auxiliary packaging plasmids (pLV2.1-GAG-POL and pLV2.1-VSVG) were co-transfected into HEK293T cells using Lipo6000™ transfection reagent (Beyotime Biotechnology). Culture supernatants containing lentiviral particles were harvested at 48 h and 72 h post-transfection, filtered through a 0.45 μm membrane, and concentrated with Lenti-X Concentrator (TaKaRa). Lentiviral titers were quantified using the Lentivirus qPCR Quantification Kit (Abcam). The volume of concentrated lentivirus required for cell transduction was determined based on viral titer and multiplicity of infection (MOI). BUMPT cells were incubated with lentivirus plus 2 μg/mL polybrene at 37 °C overnight, followed by removal of viral supernatant. At 24 h post-transduction, stable METTL3-overexpressing cell lines were selected by maintaining the cells in medium containing 2 μg/mL puromycin for 7 days.

**4. Transient Knockdown of IGF2BP3 via siRNA Transfection**

BUMPT cells were seeded 24 h prior to transfection and cultured until reaching 70–80% confluence at the time of transfection. Small interfering RNA (siRNA) specifically targeting mouse IGF2BP3 (si-IGF2BP3) and negative control siRNA (si-NC) was mixed thoroughly with Lipo8000™ transfection reagent (Beyotime Biotechnology) and transfected into BUMPT cells. Cells were harvested 48–72 h post-transfection, and the knockdown efficiency of IGF2BP3 was validated at both mRNA and protein levels.

**5. Experimental Groups and Treatment Protocol**

Six experimental groups were established as follows: ①Control group; ②Model group (TGF-β1-stimulated); ③High-dose HKSX-medicated serum (High, TGF-β1 + HKSX-medicated serum) group；④ OE METTL3 + HKSX (OE METTL3 +High, TGF-β1+HKSX-medicated serum+METTL3 overexpression) group; ⑤si IGF2BP3+HKSX (si IGF2BP3+High, TGF-β1+HKSX-medicated serum+IGF2BP3 knockdown) group; ⑥ OE METTL3+si IGF2BP3+HKSX (OE METTL3+si IGF2BP3+High, TGF-β1+HKSX-medicated serum+METTL3 overexpression+ IGF2BP3 knockdown) group.

Except for the Control group, all cells were stimulated with 5 ng/mL TGF-β1 for 24 h to establish the fibrotic injury model. Concurrently, cells in the HKSX-treated groups were cultured in medium containing 10% HKSX-medicated rat serum, while those in the other groups received an equal volume of blank rat serum as a vehicle control. After 24 h of co-incubation, cells were collected for further analysis.

Supplementary Fig
